# Complete mitochondrial genome identification of *Niwaella nigrolinea* (Cypriniformes: Cobitidae)

**DOI:** 10.1080/23802359.2022.2149247

**Published:** 2022-11-23

**Authors:** Tian-Jiang Chu, Ning Yang, Kai Liu

**Affiliations:** a Institute of Fishery Science, Hangzhou Academy of Agricultural Sciences, Hangzhou, China; b Hangzhou Reservoir Management Service Center, Hangzhou, China

**Keywords:** *Niwaella nigrolinea*, mitochondrial genome, next-generation sequencing, phylogeny

## Abstract

In this study, the complete mitochondrial genome of *Niwaella nigrolinea* (Cypriniformes: Cobitidae) from Zhejiang, China, was determined for the first time. We found that the sequenced length of the complete mitochondrial genome of *N. nigrolinea* was 16,565 bp. The genome contained 13 protein-coding genes, 22 transfer RNAs, two ribosomal RNAs, and two non-coding regions, identical to most other vertebrates. Our phylogenetic analysis results confirmed that *N. nigrolinea* was close to an unclassified *Cobitis sp*. and the fishes of the genus *Cobitis*. These data would contribute to the genetic conservation genetics and stock evaluation of *N. nigrolinea.*

Loaches of the genus *Niwaella* Nalbant, 1963 (Cypriniformes, Cobitidae) are small benthic freshwater fishes found throughout East Asia, including China, Korea, and Japan. The fish inhabits the pebble or boulder bottoms of clear, swift-moving mountain streams, lack sexual dimorphism, and have extremely elongated bodies (Chen et al. 2017). *Niwaella nigrolinea* (Chen et al. 2017) was discovered in Xin’an River, Anhui Province, China, in 2017. Chen et al. (2017) named it *N. nigrolinea*. However, at present, *N. nigrolinea* and *Cobitis nigrolinea* are considered synonyms (Perdices et al. 2016). Compared to similar species, *N. nigrolinea* has a black stripe from the occiput to the caudal fin on the dorsum, short and dense vertical bars on the dorsolateral surface, a split lower lip and mandible, undeveloped mental lobes, a thick and curved suborbital spine, and a long processus latero-caudalis and caudal peduncle (Chen et al. 2017). In addition, Chen et al. (2017) obtained 1140 bp of the mitochondrial cytochrome b gene and identified three unique haplotypes in three specimens of *N. nigrolinea*. However, to our knowledge, no publication exists on the mitochondrial genome of *N. nigrolinea*. Here, we report the complete mitochondrial genome of *N. nigrolinea* for the first time and the results of our evaluation of its phylogenetic performance among Cobitidae fishes.

*Niwaella nigrolinea* was collected from a stream beside Yunyuan Port in Chun’an County, Zhejiang Province, China (118°54′14.5′′ E, 29°49′41.5′′ N), and deposited at the National Original Breeding Farm of black Amur bream from China’s Qiantang River (120°07′21.99″E, 30°08′35.53″N). The phenol-chloroform extraction method (Green and Sambrook 2012) was applied to obtain total genomic DNA from the fin tissue deposited at Hangzhou Academy of Agricultural Sciences (http://www.hznky.com/, Kai Liu and liukai0106@email.cn) under the voucher number HXHQ202009. After quantifying the genomic DNA, it was sonicated using a Covaris M220 Ultrasonicator™ (Covaris, Inc., Woburn, MA, USA). Purified sheared DNA fragments were used to build a sequencing library and then subjected to next-generation sequencing (NGS). The NGS was performed by Origingene Bio-pharm Technology Co., Ltd. (Shanghai, China). The mitochondrial genome of *N. nigrolinea* was obtained by sequence assembly of the NGS data using NOVOPlasty ver. 4.3.1 (Dierckxsens et al. 2017). The accession number MZ707538 was assigned to the genome by GenBank. The MITOFISH prediction server (Iwasaki et al. 2013) was used to complete the annotation process.

The complete mitochondrial genome of *N. nigrolinea* from Yunyuan Port was identified to have a length of 16,565 bp and to contain 22 transfer RNAs (tRNAs), 2 ribosomal RNAs (rRNAs), 13 protein-coding genes (PCGs), and 2 non-coding regions. It is identical to those of most vertebrate mitochondrial genomes. The base composition of the mitochondrial genome of *N. nigrolinea* was found to consist of 29.39% A, 27.98% T, 25.70% C, and 16.93% G, with an AT content of 57.37%. Most PCGs and RNAs of *N. nigrolinea* are encoded on the heavy strand (H-strand) except for *ND*6 and eight tRNAs encoded on the light strand (L-strand). Apart from *CO*1, which was initiated with GTG, all 13 PCGs inside the genome had the standard start codon ATG. However, the stop codons of the 13 PCGs varied, concluding with TAG, TAA, TA − or T−. The origin of light-strand replication (OL), which could span up to 31 nucleotides, was established in the WANCY region (containing trnW, trnA, trnN, trnC, and trnY). The second non-coding region, the control region (D-loop), had a length of 912 bp and was positioned between the tRNA trnP and trnF. Figure 1, drawn by IQ-TREE ver. 2.1.3, depicts the phylogenetic tree of *N. nigrolinea*, using the 13 PCGs and the TPM2 + F + I + G4 substitution model (Minh et al. 2020). The Ultrafast Bootstrap method (repeated 1000 times) was used to test the reliability of the phylogenetic tree (Hoang et al. 2018). SH-aLRT values and Ultrafast Bootstrap values are expressed in percentages. The phylogenetic analysis showed that *N. nigrolinea* (MZ707538) initially clustered with an unclassified *Cobitis* sp. (AP013307) into a branch with a high bootstrap value. Then, they clustered with *Cobitis macrostigma* (MK156771) with a lower bootstrap value and with *Cobitis hankugensis* (MN841275) and *Cobitis elongatoides* (KF926686). However, *N. nigrolinea* (MZ707538), *C. macrostigma* (MK156771), and the other aforementioned fishes of the genus *Cobitis* were not first grouped with the other fishes of the genus *Cobitis,* including *C. choii* (EU656112), *C. sinensis* (AY526868), and *C. striata* (AB054125), but after the genus *Iksookimia* fishes. Fishes of other genera, such as *Koreocobitis*, *Paramisgurnus*, and *Misgurnus*, can be clustered in different branches from those of the genus *Cobitis.* It should be noted that *Paramisgurnus dabryanus* (KF771003) was not grouped with other *P. dabryanus* fish, and *Kichulchoia multifasciata* (KR075135) was placed in a group with fishes of the genus *Cobitis*. In addition, *Heros severus* (MT363636) representatives are in the same clade as some fishes of the genus *Cobitis*, which requires further scientific attention.

**Figure 1.**
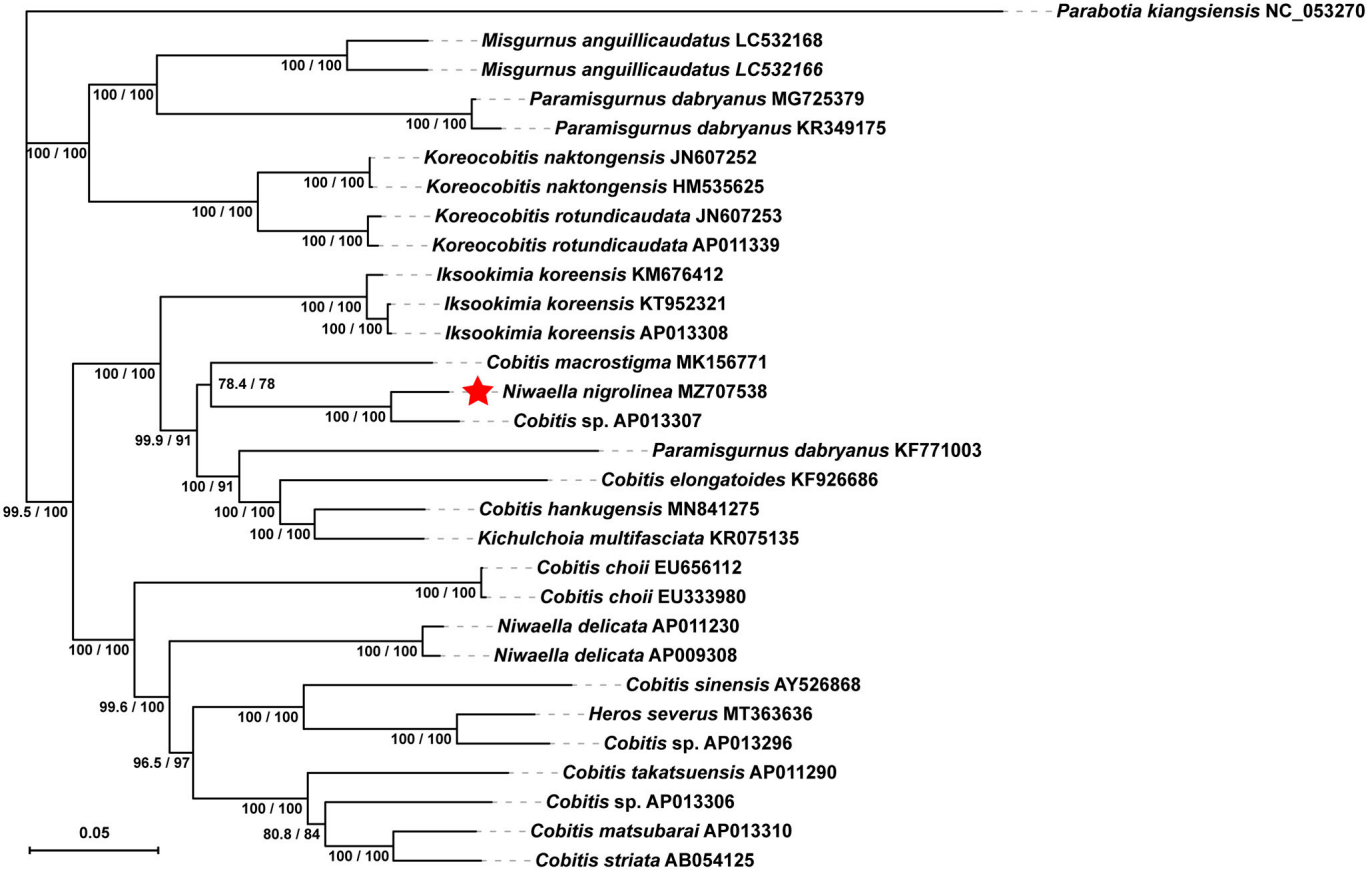
The phylogenetic tree of *Niwaella nigrolinea* was inferred using the Maximum-likelihood method based on the mitochondrial genomes. The values at each node of the tree correspond to the SH-aLRT values, and Ultrafast Bootstrap values are expressed as percentages.

## Data Availability

The data supporting this study’s findings are openly available in National Center for Biotechnology Information (NCBI) at https://www.ncbi.nlm.nih.gov/nuccore, reference number MZ707538. The associated BioProject, SRA, and BioSample numbers are PRJNA761196, SRR15734596, and SAMN21245545, respectively.
